# Molecular characterization of a potential receptor of *Eimeria acervulina* microneme protein 3 from chicken duodenal epithelial cells

**DOI:** 10.1051/parasite/2020014

**Published:** 2020-03-20

**Authors:** Zhenchao Zhang, Zhouyang Zhou, Jianmei Huang, Xiaoting Sun, Muhammad Haseeb, Shakeel Ahmed, Muhammad Ali A. Shah, Ruofeng Yan, Xiaokai Song, Lixin Xu, Xiangrui Li

**Affiliations:** 1 MOE Joint International Research Laboratory of Animal Health and Food Safety, College of Veterinary Medicine, Nanjing Agricultural University Nanjing 210095 PR China; 2 School of Basic Medical Sciences, Xinxiang Medical University Xinxiang 453003 Henan PR China; 3 Department of Pathobiology, Faculty of Veterinary & Animal Sciences, Pir Mehr Ali Shah Arid Agriculture University 46000 Rawalpindi Pakistan

**Keywords:** *Eimeria acervulina*, EaMIC3, Receptor, Ubiquitin conjugating enzyme E2F, Chicken

## Abstract

*Eimeria acervulina* is one of seven *Eimeria* spp. that can infect chicken duodenal epithelial cells. *Eimeria* microneme protein 3 (MIC3) plays a vital role in the invasion of host epithelial tissue by the parasite. In this study, we found that chicken (*Gallus gallus*) ubiquitin conjugating enzyme E2F (UBE2F) could bind to the MIC3 protein of *E. acervulina* (EaMIC3), as screened using the yeast two-hybrid system, and that it might be the putative receptor protein of EaMIC3. The UBE2F gene was cloned from chicken duodenal epithelial cells. The recombinant protein of UBE2F (rUBE2F) was expressed in *E. coli* and the reactogenicity of rUBE2F was analyzed by Western blot. Gene sequencing revealed that the opening reading frame (ORF) of UBE2F was 558 base pairs and encoded a protein of 186 amino acids with a molecular weight of 20.46 kDa. The predicted UBE2F protein did not contain signal peptides or a transmembrane region, but had multiple O-glycosylation and phosphorylation sites. A phylogenetic analysis showed that the chicken UBE2F protein is closely related to those of quail and pigeon *(Coturnix japonica* and *Columba livia)*. A sporozoite invasion-blocking assay showed that antisera against rUBE2F significantly inhibited the invasion of *E. acervulina* sporozoites *in vitro*. Animal experiments indicated that the antisera could significantly enhance average body weight gains and reduce mean lesion scores following a challenge with *E. acervulina*. These results therefore imply that the chicken UBE2F protein might be the target receptor molecule of EaMIC3 that is involved in *E. acervulina* invasion.

## Abstract (fr)

*Eimeria acervulina* est l’une des sept *Eimeria* spp. qui peuvent infecter les cellules épithéliales duodénales de poulet. La protéine 3 du micronème d’*Eimeria* (MIC3) joue un rôle vital dans l’invasion du tissu épithélial de l’hôte par le parasite. Dans cette étude, nous avons constaté que l’enzyme de conjugaison de l’ubiquitine de poulet E2F (UBE2F) pouvait se lier à la protéine MIC3 d’*E. acervulina* (EaMIC3), telle que testé à l’aide du système de levure à deux hybrides, et qu’il pourrait s’agir de la protéine réceptrice putative d’*EaMIC3*. Le gène UBE2F a été cloné à partir de cellules épithéliales duodénales de poulet. La protéine recombinante d’UBE2F (rUBE2F) a été exprimée dans *E. coli* et la réactogénicité de rUBE2F a été analysée par Western blot. Le séquençage génétique a révélé que le cadre de lecture d’ouverture (ORF) d’UBE2F était de 558 paires de bases et codait une protéine de 186 acides aminés avec un poids moléculaire de 20,46 kDa. La protéine UBE2F prédite ne contenait pas de peptides signaux ni de région transmembranaire, mais avait plusieurs sites d’O-glycosylation et de phosphorylation. Une analyse phylogénétique a montré que la protéine UBE2F de poulet est étroitement liée à celles de la caille et du pigeon (*Coturnix japonica* et *Columba livia*). Un test de blocage des invasions de sporozoïtes a montré que les antisérums dirigés contre rUBE2F inhibaient de manière significative l’invasion des sporozoïtes d’*E. acervulina* in vitro. Les expériences sur les animaux ont indiqué que les antisérums pourraient améliorer de manière significative les gains de poids corporel moyens et réduire les scores moyens de lésions suite à une infection avec *E. acervulina*. Ces résultats impliquent donc que la protéine UBE2F de poulet pourrait être la molécule de récepteur cible d’EaMIC3 impliquée dans l’invasion d’*E. acervulina*.

## Introduction

Avian coccidiosis is caused by intestinal infection with single or multiple *Eimeria* spp. and results in huge production losses globally [14, 22]. *Eimeria acervulina* is one of seven *Eimeria* spp., and it infects chicken duodenal epithelial cells resulting in malabsorption, poor feed utilization, and reduced body weight gains (BWGs) [33, 35].

*Eimeria* spp. are site-specific when invading and reproducing in the chicken intestine. For instance, *Eimeria tenella* infects the caecum, *Eimeria acervulina* infects the duodenum, and *Eimeria maxima* infects the jejunum [17]. However, to date, the molecular mechanisms of invasion and the site-specificity of *Eimeria* spp. have not been elucidated.

Recently, it has been reported that molecules on the surface of intestinal epithelial cells, which act as receptors or recognition sites for sporozoite invasion, result in the invasion and site specificity [1, 7]. Furthermore, it has been confirmed that EtMIC3 of *E. tenella* plays a key role in invasion and site specificity [21]. It has also been reported that *E. acervulina* MIC3 (EaMIC3) and *E. mitis* (EmMIC3) are expressed in the sporozoite and merozoite stages, localized at the parasite apex, and could significantly protect chickens from *E. acervulina* infection [14, 36]. These findings show that the *Eimeria* MIC3 proteins are the key molecules associated with invasion and site specificity.

However, no studies regarding *E. acervulina* invasion receptors have been reported. In the current study, the ubiquitin conjugating enzyme E2F (UBE2F) protein of chicken duodenal epithelial cells was identified to potentially interact with EaMIC3, as screened using the yeast two-hybrid system. Furthermore, the UBE2F gene was obtained by PCR amplification and expressed in a prokaryotic expression system. Invasion inhibition by antiserum against rUBE2F on *E. acervulina* sporozoites was observed through sporozoite invasion-blocking assays and chicken challenge experiments.

## Materials and methods

### Ethics approval

The study was reviewed and approved by the Science and Technology Agency of Jiangsu Province. The approval ID is SYXK (SU) 2010–0005.

### Experimental chickens and parasites

*Eimeria-free* Hy-Line layer one-day-old chicks were provided with *ad libitum* feed and water without anticoccidial drugs.

*Eimeria acervulina*, Jiangsu strain, was reproduced and maintained in the Laboratory of Veterinary Parasite Disease, Nanjing Agricultural University, China.

Sporozoites from *E. acervulina* oocysts were purified on DE-52 anion-exchange columns using a previously described protocol [34].

### Isolation and identification of chicken duodenal epithelial cells

The duodenal epithelial cells of two-week-old chicks were isolated as previously described [34]. Coccidian-free chicks were emerged in 70% ethanol after they were killed by exsanguination. Five minutes later, the duodenums were dissected using scissors and placed into Hanks’ balanced salt solution (HBSS; PAA Laboratories, Linz, Austria). Subsequently, the duodenums were washed with HBSS until the mucus was completely removed. Following dissection of the mucosa into small strips (3 × 20 mm^2^), the strips were placed into 1 mM DTT (Sigma–Aldrich, Taufkirchen, Germany) in 50 mL HBSS (30 min at ambient temperature). Sequentially, the mucosal strips were incubated in 1 mM EDTA (Sigma) for 10 min at 37 °C. Mucosal strips were briefly rinsed in HBSS to eliminate already detached duodenal epithelial cells and transferred to fresh HBSS at ambient temperature, followed by 5–10 vigorous shakes of the container. This procedure led to instant detachment of duodenal epithelial cells in a full-length crypt formation. After rapid removal of the mucosal strips by passing the solution over a coarse mesh (400 μm, Rotilabo sieve; Carl Roth GmbH, Karlsruhe, Germany), rapid purification of detached duodenal epithelial cells was achieved using a mesh filter (80 μm pore size; Sefar, Kansas City, KS, USA) fixed with tape to a plastic ring (5 cm diameter, 2 cm height, and 3 mm thickness). The suspension containing duodenal epithelial cell crypts was gently but rapidly passed over the mesh to separate the cell crypts from single cells (erythrocytes, leukocytes, fibroblasts, etc.), which easily passed through the filter. The filter was then rapidly inverted, and purified intact duodenal epithelial cell crypts were immediately washed out with Dulbecco’s Modified Eagle Medium (DMEM) (Gibo^®^, Life Technologies, MD, USA) at ambient temperature. The duodenal epithelial cell crypt solution was then rapidly transferred to an ECM-coated culture dish and cultured at 41 °C and 5% CO_2_ for 1.5 h. The non-adherent cells were collected for identification of the duodenal epithelial cells and construction of a cDNA library.

Duodenal epithelial cells were identified by cell alkaline phosphatase (cAKP) stain (Azo-coupling method). The separated duodenal epithelial cells were fixed on a polylysine-coated cover slip and the slip was washed three times with 0.1 M PBS (pH 7.2). The duodenal epithelial cells were stained using a cAKP kit (JianCheng, Nanjing, China), according to the manufacturer’s instructions.

### RNA extraction

Total RNA was extracted from the *E. acervulina* sporozoites and the chicken intestinal epithelial cells using an E.Z.N.A.^®^ Total RNA Maxi Kit (OMEGA, Norcross, GA, USA), according to the manufacturer’s instructions. The quantity of RNA was estimated by spectrophotometry and samples with a ratio OD260/OD280 between 1.9 and 2 were used.

### Construction of the bait vector and the cDNA library of chicken duodenal epithelial cells

The ORF of EaMIC3 (GenBank accession no. KU359773) was cloned from the *E. acervulina* sporozoite RNA by reverse transcription PCR (*Sfi* I anchored forward primer: 5′ – GCATGGCCATTACGGCCATGCCTGTATATGCGAGATACGACG – 3′; *Sfi* I anchored reverse primer: 5′ – CGACGGCCGCCTCGGCCTTGCCGATGCACGTGAACTTT – 3′) and inserted into bait vector pDHB1 to form pDHB1–EaMIC3.

RNase-free DNase I (TaKaRa, Clontech Laboratories, CA, USA) was used to remove the genomic DNA contamination in the prepared RNA samples. Subsequently, a SMART cDNA Library Construction Kit (TaKaRa, Clontech Laboratories, CA, USA) was used to reverse transcribe RNA into double-stranded cDNA, according to the manufacturer’s instructions. The double-stranded cDNA was normalized using a Trimmer-2 cDNA normalization kit (Evrogen, Moscow, Russia), according to the manufacturer’s instructions. A MiniBest DNA Fragment Purification Kit (TaKaRa) was used to purify cDNA, according to the manufacturer’s instructions. CHROMA SPINTM-1000 (Clontech Laboratories, CA, USA) was used to select the cDNA greater than 0.5 kb. The cDNA library was created by using a SMART cDNA Library Construction Kit (TaKaRa, Clontech Laboratories) and cloned into the pray plasmid pPR3-N. To confirm the size of clone inserts, plasmid DNA was extracted from 32 clones randomly and digested using restriction enzyme *Sfi* I. Ninety-six monoclones were randomly selected for analysis of homogenization by sequencing.

### Identification of binding partners for EaMIC3 using yeast two-hybrid (YTH) screening

A DUALhunter starter kit (Dualsystems Biotech, Schlieren, Switzerland) was used to identify the EaMIC3 binding molecule from chicken duodenal epithelial cells by YTH screening [6]. The bait plasmid pDHB1–EaMIC3 was transformed into yeast NMY51. After confirming the expression of the bait and functional assay and optimizing the screening stringency, the plasmid pDHB1–EaMIC3 was used to screen a chicken duodenal epithelial cell cDNA library to identify the proteins interacting with EaMIC3. Positive colonies were selected and the plasmids were extracted using a Yeast Plasmid Extraction Kit (OmegaBio-tek, Norcross, GA, USA). The selected prey plasmids were transformed into *E. coli* DH5α and recovered by ampicillin selection. The pPR3N-F and pPR3N-R primers were used to detect the inserted fragments in the selected prey plasmid gene using PCR. Then the isolated positive prey plasmids were retransformed into yeast NMY51, which contained the bait plasmid pDHB1–EaMIC3 to eliminate false positives. LargeT was used as a bait control and Alg5 fused to NubG or NubI was used as the negative or positive prey control, respectively. The inserted fragments in these prey plasmids were sequenced and the DNA sequences were used to search GenBank.

### Cloning of the UBE2F gene

Total RNA of chicken intestinal epithelial cells was reverse transcribed into cDNA as a template. Specific primers were designed and synthetized to amplify the ORF of UBE2F (E*coR* I anchored forward primer: 5′ – CGGAATTCTGCTCACTCTGGCAAGCAA – 3′; *Xh* I anchored reverse primer: 5′ – CCCTCGAGTCATCTCGCGTAGCGCTTAA – 3′). The amplified UBE2F gene was ligated with pMD-19T cloning vector (TaKaRa, Dalian, China) and transformed into *E. coli* DH5α competent cells (Vazyme Biotech Co., Ltd., Nanjing, China). Subsequently, clones of UBE2F were checked by sequence confirmation through the online database (https://blast.ncbi.nlm.nih.gov/Blast.cgi).

### Sequence analysis

Sequence similarity was checked through the online database BLASTP (https://blast.ncbi.nlm.nih.gov/Blast.cgi). LaserGene (DNAStar, Madison, WI, USA) was used to predict the protein isoelectric point (pI) of the UBE2F protein. The signal peptide, transmembrane region, glycosylation, phosphorylation, and GPI modification sites of the UBE2F protein were analyzed using the on-line prediction service CBS Prediction Servers (http://www.cbs.dtu.dk/services/). UBE2F protein sequences were aligned using CLUSTALW1.8 (http://www.ebi.ac.uk/clustalw).

### Expression and purification of recombinant UBE2F protein

The identified recombinant plasmid pMD-19T-UBE2F was digested by endonuclease E*coR* I and *Xho* I. Subsequently, the target fragment was inserted into the pET-32a expression vector and transformed into *E. coli* BL21 (DE3) competent cells (Vazyme biotech Co., Ltd., Nanjing, China). Positive clones were selected and identified by PCR, endonuclease digestion, and DNA sequencing. The recombinant UBE2F protein (rUBE2F) was expressed in *E. coli* BL21 and purified using a Ni^2+^‐nitrilotriacetic acid (Ni-NTA) column (GE Healthcare, Chicago, IL, USA). The purified protein was determined using 12% sodium dodecyl sulfate polyacrylamide gel electrophoresis (SDS-PAGE) and the concentration of the rUBE2F was determined by the Bradford procedure using bovine serum albumin (BSA) as a standard. The purified protein was stored at −70 °C until use. Meanwhile, the pET-32a fusion protein was prepared by the same method.

### Preparation of chicken antiserum against rUBE2F

To generate chicken antiserum against rUBE2F, two-week-old chicks were vaccinated with 200 μg purified rUBE2F by intramuscular injection into their thighs and the chicks were given four booster vaccinations at intervals of seven days. Finally, the antiserum was collected and stored at −70 °C. Chick antiserum against the pET-32a fusion protein was prepared by the same method and negative chick serum was collected simultaneously. The antibody titer was determined by ELISA.

### Western blot analysis of rUBE2F [15]

The rUBE2F was separated by SDS-PAGE and transferred to polyvinylidene fluoride membranes (Bio-Rad, Hercules, CA, USA). The membranes were blocked in TBS (Tris-buffer saline)-Tween 20 (TBST) containing 2% BSA and then incubated with chicken antisera (1:100) for 1 h at 37 °C. After five washes with TBST, the membranes were incubated with horseradish peroxidase (HRP)-conjugated goat anti-chicken IgG (1:3000, Sigma–Aldrich) for 1 h at 37 °C. The bound antibodies were revealed with 3, 30-diaminobenzidine (DAB; Boster Biotechnology, Wuhan, China), according to the manufacturer’s instructions

### Evaluation of the invasion inhibition of anti-rUBE2F serum *in vitro*

To evaluate the inhibitive effects of anti-rUBE2F serum on the invasion of *E. acervulina in vitro*, the sporozoites from *E. acervulina* oocysts were cleaned and sporulated, as previously described [18]. Two-week-old chicks were randomly divided into five groups of five. The duodenal sections (5 cm lengths) were collected and preserved in preheated HBSS at 41 °C. One end of each duodenum section was ligated and 1.0 × 10^6^ *E. acervulina* sporozoites were used to infect the section. Meanwhile, chicken anti-rUBE2F serum was diluted with PBS at a ratio of 1:5 and was added into the duodenum sections and mixed with the sporozoites, followed by ligation of the second end of the section. Chicken antiserum against the pET-32a protein and the chicken negative serum at the same dilution were used as controls, following the same method. Duodenums were then incubated in preheated PBS at 41 °C. After 20 min, the effluents were collected by washing the sections with PBS. Sporozoites in the effluents were counted and the invasion inhibition rates of the antisera were calculated using the following equation:

Sporozoite invasion inhibition rate=number of sporozoites in the effluent/total number of infected sporozoites×100%.

### Protective effects of anti-rUBE2F serum on chicks challenged with *E. acervulina*

Four-week-old chicks of similar weight were randomly divided into five groups of 15. Each group, with the exception of the unchallenged control group, were infected with 1.2 × 10^5^ *E. acervulina* sporulated oocysts by oral gavage. The unchallenged control chicks were given the same volume of PBS by oral gavage. At the same time, 0.1 mL of chicken antiserum against rUBE2F diluted with PBS at a ratio of 1:5 was injected intravenously into the wings of the experimental group once a day for six days [8, 31]. The chicken antiserum against pET-32a vector protein and the chicken negative serum were injected by the same method, as the control groups. On Day 7, all the chicks were humanely killed and body weight gains and lesion scores were evaluated. Chick enteric contents were collected separately to evaluate the number of oocysts per gram feces (OPG) using a McMaster counting chamber, as previously described [36].

### Statistical analysis

One-way analysis of variance (ANOVA) with Duncan’s multiple range tests were used for the determination of statistical significance by using the SPSS statistical package (SPSS for Windows 16, SPSS Inc., Chicago, IL, USA). Differences among groups were tested and *p* < 0.05 was considered to indicate a significant difference.

## Results

### Identification of the bait vector and the cDNA library of chicken duodenal epithelial cells

**Figure 1:**
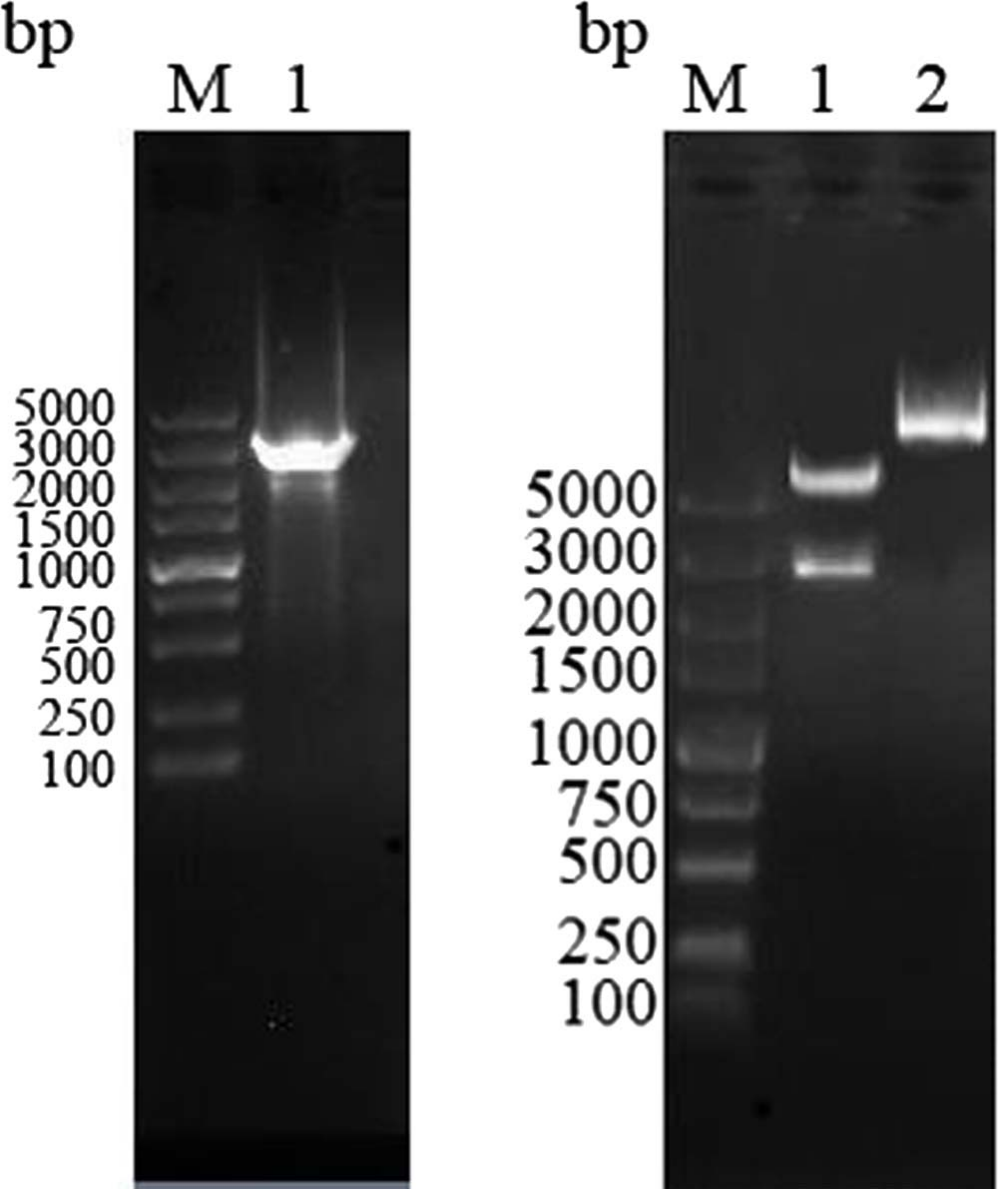
Construction and restriction endonuclease analysis of recombinant bait plasmid pDHB1–EaMIC3. (A) M: DL5000 marker; 1: The product of EaMIC3 PCR. (B) M: DL5000 marker; 1: pDHB1–EaMIC3 digested by *Sfi* I; 2: pDHB1–EaMIC3.

The EaMIC3 gene in bait vector pDHB1–EaMIC3 was obtained by RT-PCR and the target fragment size of 2607 bp (Fig. 1A) was confirmed through restriction enzyme digestion (Fig. 1B). Sequence analysis also confirmed the insert was EaMIC3 ORF, indicating successful construction of the bait vector pDHB1–EaMIC3.

**Figure 2:**
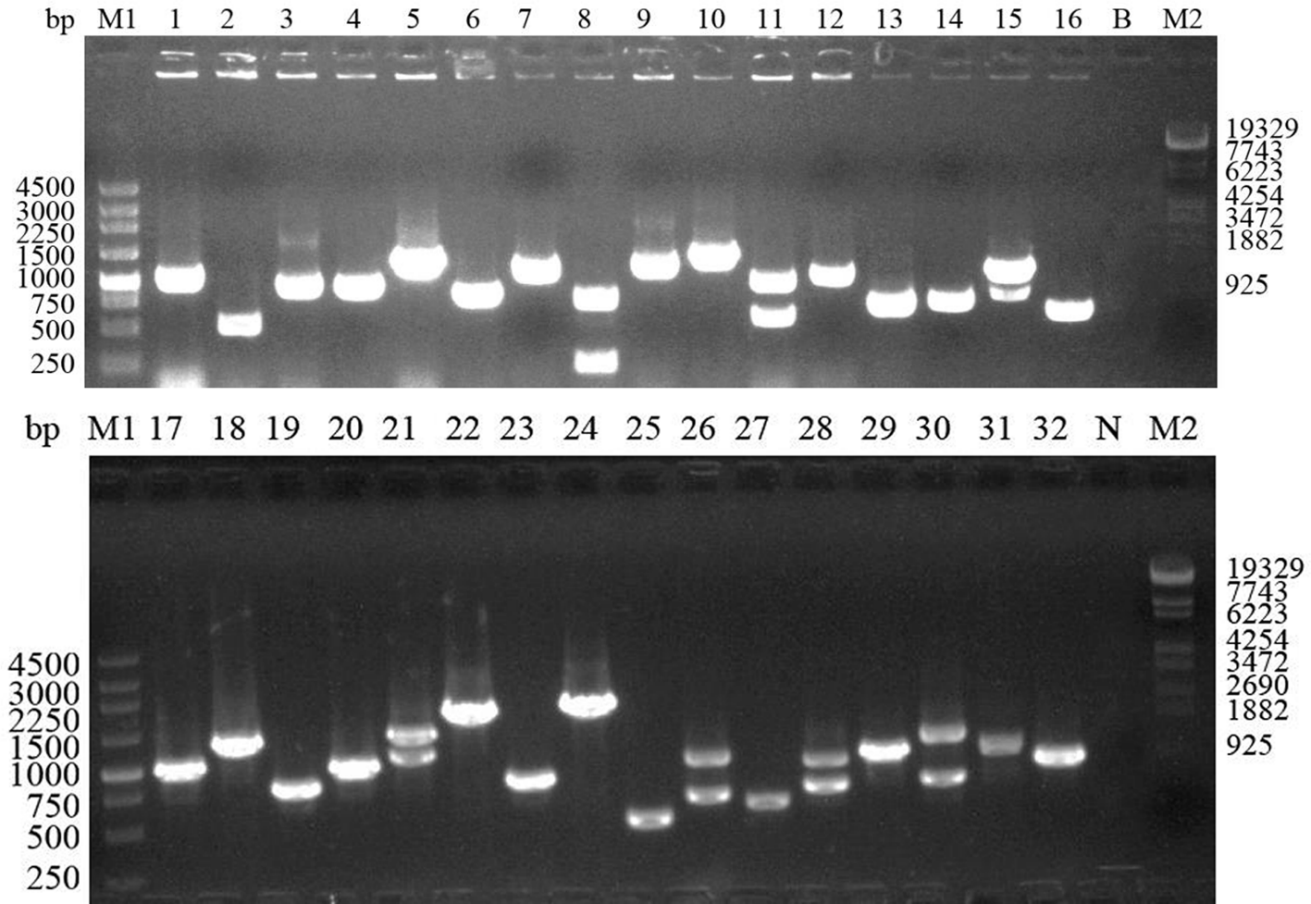
Analysis of the inserted fragment in chicken duodenal epithelial cell normalized cDNA library using PCR. M1: DL19329 marker; M2: DL4500 marker; 1–32: PCR analysis of 32 bacterial colonies; B: Blank control without template; N: Negative control of library vector.

The chick duodenal epithelial cells were isolated, identified by cAKP staining, and used to construct a cDNA library. The cDNA library contained at least 4 × 10^6^ primary recombinants, and the average insert size was 1.0 kb (Fig. 2). In addition, 96 monoclones were randomly selected for sequencing and the results showed that 0 of 96 monoclones were redundant, indicating good homogenization.

### UBE2F is a binding protein for EaMIC3

In the YTH screening, 37 clones encoding proteins that showed a potential interaction with the EaMIC3 proteins in yeast cells were identified. Multiple potential EaMIC3-interacting proteins were identified in the retest, and 17 clones were obtained. These genes were then identified by DNA sequencing and searching of GenBank. One gene was determined to be UBE2F (NCBI accession number XM_013178750.1).

### Cloning of the UBE2F gene and sequence analysis of UBE2F

**Figure 3:**
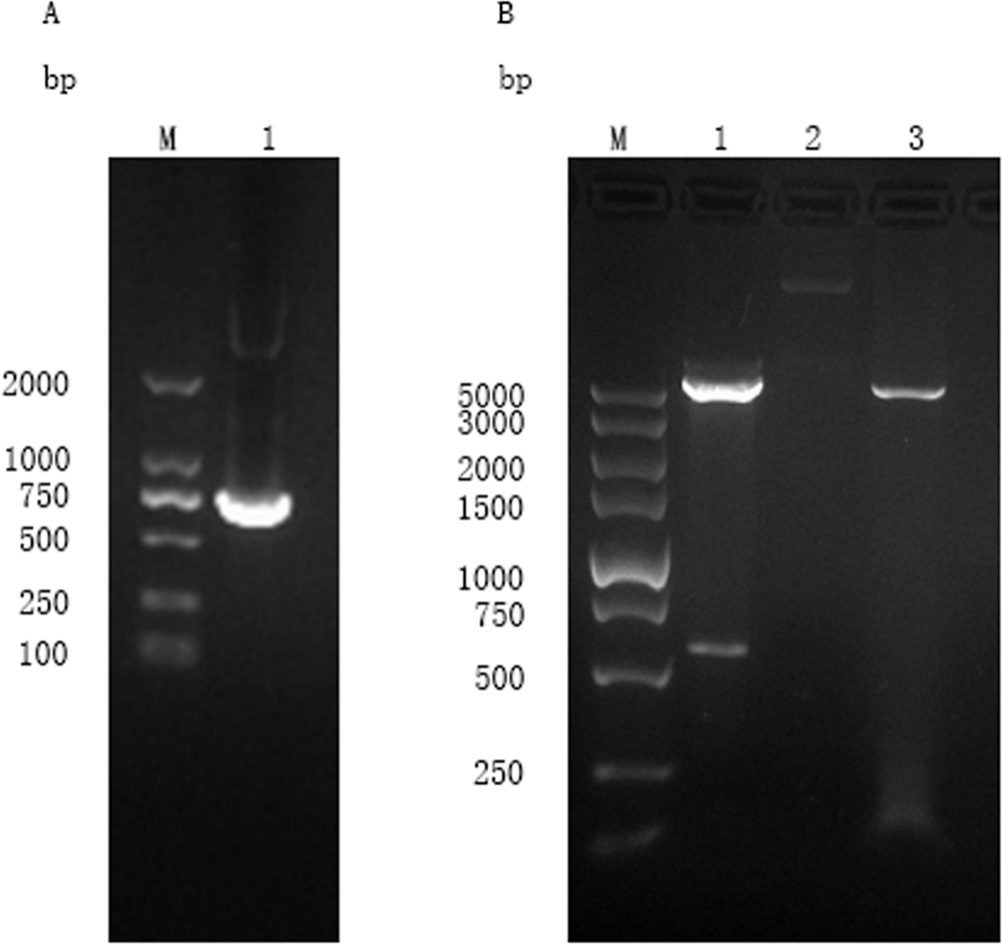
Agarose gel electrophoresis of UBE2F ORF and identification of recombinant plasmid pET-32a-UBE2F digested by E*coR* I and *Xho* I. (A) (Lane M) DNA molecular weight marker DL 2000 (ordinate values in bp); (Lane 1) the ORF of UBE2F. (B) (Lane M) DNA molecular weight marker DL 5000 (ordinate values in bp); (Lane 1) the recombinant plasmid pET-32a-UBE2F digested by E*coR* I and *Xho* I; (Lane 2) the recombinant plasmid pET-32a-UBE2F; (Lane 3) the plasmid of pET-32a (+) vector digested by E*coR* I and *Xho* I.

**Figure 4:**
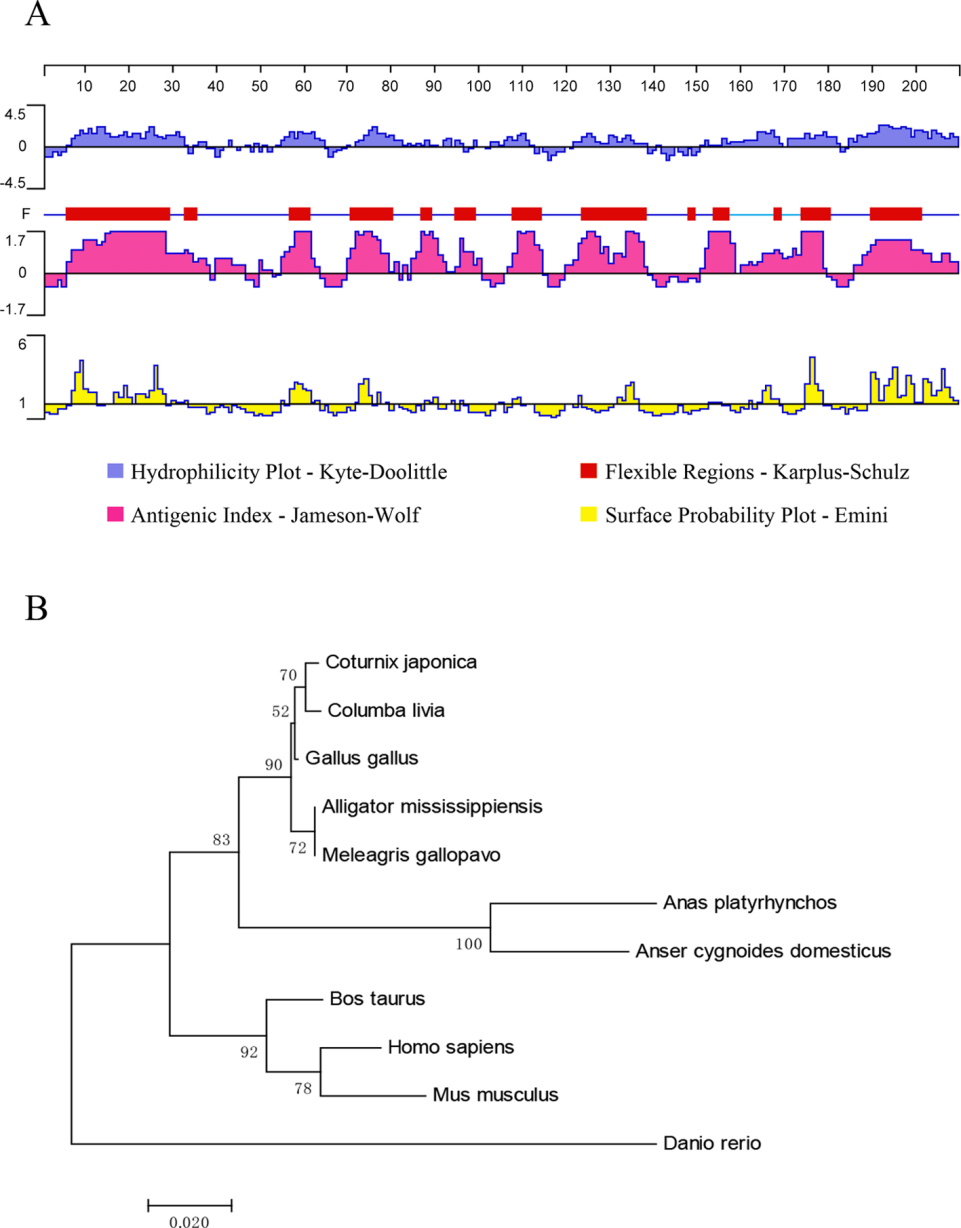
(A) Linear-B cell epitopes of UBE2F predicted by DNASTAR are shown in hydrophilicity plots, flexible regions, antigenic indices, and surface probability rules. (B) The phylogenetic tree of amino acid sequences of UBE2F in poultry and wildfowl.

The ORF of UBE2F in plasmid pET-32a-UBE2F was obtained by RT-PCR (Fig. 3A), and a target fragment with a size of 558 bp (Fig. 3B) was identified by enzymatic digestion. Sequence analysis showed that the vector insert was the ORF of UBE2F. This result indicated that the prokaryotic expression vector pET-32a-UBE2F was constructed correctly. The ORF was predicted to encode a 186-amino acid protein with a molecular weight of 20.5 kDa and a pI of 6.50. The predicted UBE2F protein did not contain a signal peptide or transmembrane region. One N-glycosylation site, four O-glycosylation, and 19 phosphorylation sites were found in the predicted protein, but no GPI anchor could be detected. As shown in Figure 4A, the protein had six hydrophilic regions including 6–33, 54–64, 72–86, 103–112, 122–138, and 151–186, and six high antigenic indices and consecutive regions including 6–46, 55–63, 70–100, 107–114, 120–138, and 151–186. Moreover, most regions of the UBE2F protein were hydrophilic plots and flexible regions. The phylogenetic tree of amino acid sequences was built using MEGA4.0 (https://www.megasoftware.net/) and the cladogram result (Fig. 4B) showed that kinship of the UBE2F protein was lowly related in poultry and wildfowl.

### Expression and purification of rUBE2F and pET-32a proteins

**Figure 5:**
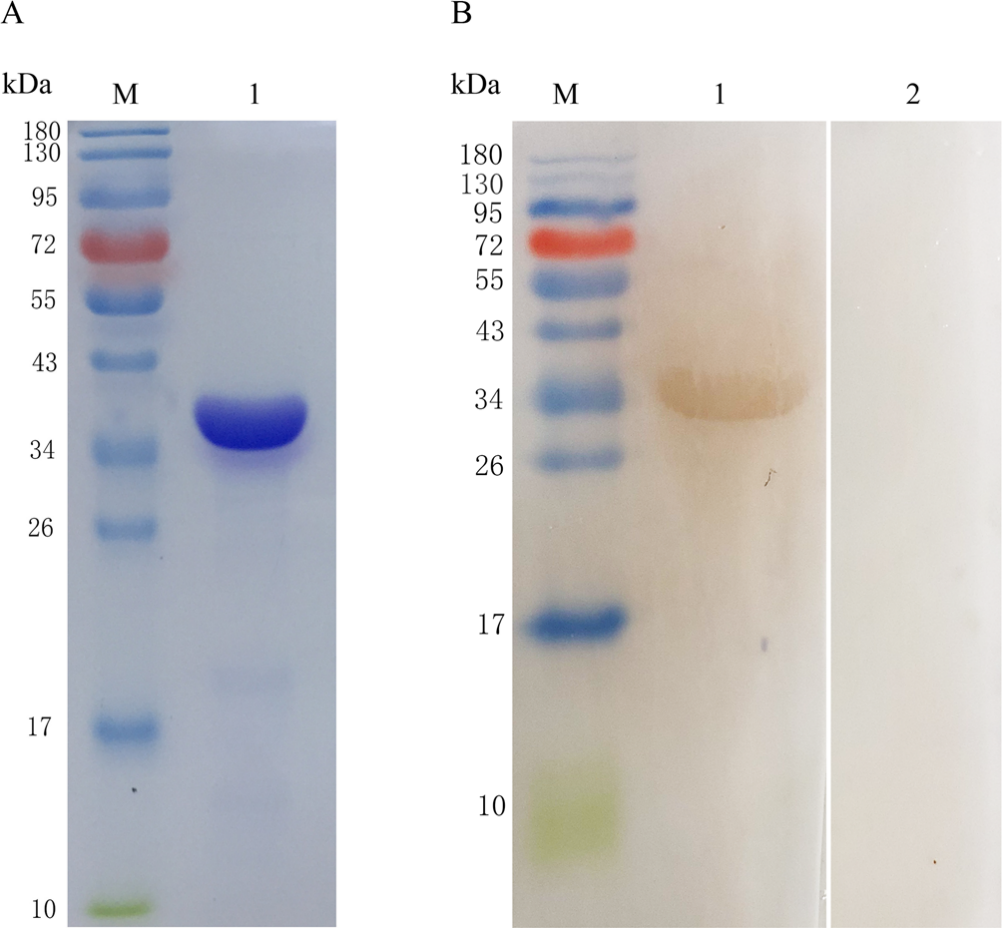
(A) Expression and purification of UBE2F. (Lane M) protein Mark (ordinate values in kDa); (Lane 1) recombinant UBE2F purified through an Ni-NTA column. (B) Western blot analysis of recombinant UBE2F using DAB. (Lane M) protein Mark (ordinate values in kDa); (Lane 1) recombinant UBE2F recognized by anti-rUBE2F chicken serum; (Lane 2) negative serum control.

The rUBE2F was expressed in *E. coli* BL21 (DE3) and purified in a Ni-NTA column. The size of rUBE2F was consistent with the molecular weight sum of fusion protein of the pET-32a vector (18 kDa) and UBE2F (21 kDa) and exhibited a single band in SDS-PAGE gel with a molecular weight of around 39 kDa (Fig. 5A).

### Immunoblot for the recombinant protein

Western blot showed that the rUBE2F could be recognized by anti-rUBE2F chicken serum, but could not be recognized by normal chicken serum (Fig. 5B). The antibody titer of chicken anti-rUBE2F was 2^10^, and this could be used for subsequent research.

### Inhibition of sporozoite invasion by antisera against rUBE2F *in vitro*

**Figure 6:**
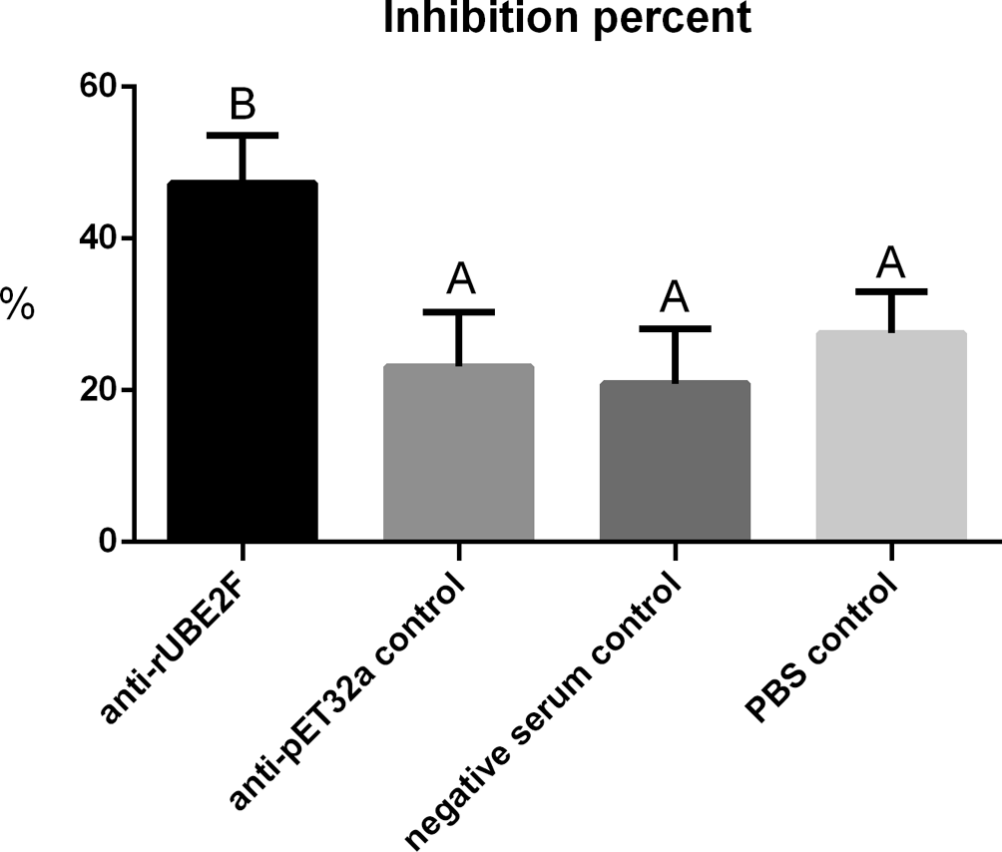
The inhibition of *Eimeria acervulina* sporozoite invasion by chicken antisera against rUBE2F. The inhibitive ratio was calculated and expressed as mean ± *SD*. In each column there is a significant difference (*p* < 0.01) between means and ranks with different letters, and no significant difference (*p* > 0.05) between means and ranks with the same letter.

The *in vitro* inhibition of *E. acervulina* sporozoite invasion by antisera against rUBE2F is shown in Figure 6. As compared with anti-pET-32a, negative serum, and the PBS control group, the anti-UBE2F group significantly reduced the efficiency of *E. acervulina* sporozoite invasion (*p* < 0.01). No significant differences were observed among anti-pET32a, negative serum, and PBS control groups (*p* > 0.05). These results indicate that antiserum against rUBE2F was effective in inhibiting the invasion of *E. acervulina* sporozoites in the duodenum *in vitro*.

### The protective efficacy of antisera against rUBE2F in challenged chicks

The efficacy of invasion inhibition of antiserum against rUBE2F is shown in Table 1. No chicks died following the *E. acervulina* challenge in any group during the experimental trial. As compared with anti-pET32a protein, negative serum, and the challenged control group, chicks in the anti-rUBE2F group showed significantly increased body weight gains and decreased lesion scores (*p* < 0.01). No significant differences were observed between the anti-UBE2F and the anti-pET32a protein, negative serum, and challenged control group regarding oocyst output (*p* > 0.05). These results indicate that antiserum against rUBE2F partially mitigate invasion inhibition against an infection challenge by *E. acervulina*.

**Table 1:** Effects of antiserum against rUBE2F during *E. acervulina* challenge on different parameters.

Groups	Average body weight gains (g)	Mean lesion scores	Oocyst output (Ig)
Unchallenged control	90.46 ± 7.97^A^	0.00 ± 0.00^A^	–^A^
Anti-pET32a protein control	19.36 ± 3.96^C^	2.35 ± 0.93^C^	6.30 ± 0.23^B^
Negative serum control	21.43 ± 4.33^C^	2.31 ± 0.81^C^	6.30 ± 0.33^B^
Anti-rUBE2F	39.40 ± 11.86^B^	1.37 ± 0.83^B^	6.13 ± 0.24^B^
Challenged control	18.21 ± 9.29^C^	2.74 ± 0.93^C^	6.28 ± 0.35^B^

Significant difference (*p* < 0.01) between means and ranks with different letters, and no significant difference (*p* > 0.05) between means and ranks with the same letter; the oocyst output was zero (designated as “–”) in the unchallenged control group.

## Discussion

Coccidiosis is a deadly disease that hampers the productivity and welfare of commercial chicken enterprises. Thus, the disease is a major threat to the global poultry industry [12, 30, 32, 37]. Seven known species of *Eimeria* cause coccidiosis in chickens by affecting the different parts of the intestinal tract in a site-specific manner [10, 23]. Growing evidence indicates that molecular interactions between *Eimeria* sporozoites and host cells provide a prelude to, and result in, site-specific invasion [1].

It is suggested that the proteins secreted from apicomplexan microneme organelles (MICs) of the sporozoites allow parasites to bind a diverse range of host cell oligosaccharide epitopes and play important roles in parasite adhesion to and invasion of host cells [5]. In *Toxoplasma gondii* and *E. tenella*, the sialic-acid binding MAR (microneme adhesive repeat) domain in the MICs was shown to make a significant contribution to different host and tissue tropisms [25]. The dual immunofluorescence staining of *E. tenella* microneme 3 (EtMIC3) and 5 (EtMIC5) on fixed and permeabilized sporozoites of *E. tenella* showed that EtMIC3 was located mainly at the apical tip of the sporozoite, while the majority of EtMIC5 labeling was detected just posterior to this region [8]. Moreover, EtMIC3 could bind to sialic acid-bearing molecules on the epithelial cell surface of the host, and played a key role in sporozoite invasion [19, 20].

*Eimeria acervulina* infects the duodenal epithelium of chickens, which results in morphological and functional damage, leading to a reduction in nutrient digestion and growth performance in broilers [9]. Previous research has shown that EaMIC3 is expressed in the sporozoite and merozoite stages of *E. acervulina* and could protect chickens from *E. acervulina* infection [36]. Thus, it might also play an important role in the specificity of invasive and parasitic sites [1, 28].

Although many invasion-related molecules of *Eimeria* have been studied [13, 25], there are only a few reports concerning sporozoite receptors on host epithelia. In the current study, a cDNA library of chicken duodenal epithelial cells was constructed and screened for EaMIC3 receptor molecules by YTH. Our results show that the UBE2F from chick duodenal epithelial cells could interact with EaMIC3 and that antiserum against rUBE2F significantly inhibited the invasion of *E. acervulina* sporozoites *in vitro*, and could significantly enhance average BWGs and reduce mean lesion scores after a challenge with *E. acervulina in vivo*. These results suggest that the chicken protein UBE2F might be the target receptor molecule of EaMIC3 involved in the invasion of *E. acervulina*.

Ubiquitin-conjugating enzymes, also known as E2 enzymes and as ubiquitin-carrier enzymes, perform the second step in the ubiquitination reaction that targets a protein for degradation via the proteasome [2, 26]. UBE2F plays a specific role in the regulation of ubiquitin chain assembly and topology and the initiation or elongation of a ubiquitin chain [16, 24, 27]. Interaction between EaMIC3 and UBE2F might induce ubiquitination of membrane proteins in host cells, leading to cell breakdown, thus achieving *E. acervulina* sporozoite invasion into host cells and pathogenesis [11].

In the current study, the complete gene sequence of chicken UBE2F was successfully obtained using PCR. The nucleic acid sequence of UBE2F showed that it contained a 558 bp ORF encoding a protein of 186 amino acids. The molecular mass of the deduced translation product was about 20.5 kDa. The predicted UBE2F protein did not contain a signal peptide or transmembrane region but did contain multiple O-glycosylation and phosphorylation sites. The modulation of glycosylation and phosphorylation to proteins is required for physiological functions. The process of O-glycosylation involves the addition of N-acetyl-galactosamine to serine or threonine residues by N-acetylgalactosaminyltransferase, followed by other carbohydrates such as galactose and sialic acid [29]. EtMIC3 has high specificity for sialylated glycan, and it contains several sialic-acid binding MARs. The presence of multiple O-glycosylation and phosphorylation sites in UBE2F indicated that sialic acids could be added to UBE2F by O-glycosylation and phosphorylation; this supports the conjecture that UBE2F is the receptor of EaMIC3 involved in the invasion of *E. acervulina*.

The salivary glands of *Aedes aegypti* mosquitoes contain the receptor of the *malaria* sporozoite, and antiserum against the salivary gland could block sporozoite invasion *in vivo* [3]. Monoclonal antibodies against sporozoite receptors could also inhibit the invasion of salivary glands by *Plasmodium yoelii* [4]. In this study, antiserum against rUBE2F significantly inhibited *E. acervulina* sporozoite invasion *in vitro* and *in vivo*. These results suggest that UBE2F plays an important role as the EaMIC3 receptor in *E. acervulina* invasion into host cells. However, the antiserum against rUBE2F did not completely inhibit *in vitro* and *in vivo* invasion, which suggests that there might be other molecules involved in the invasion of *E. acervulina* into host cells, or that the antiserum dose was insufficient. This needs to be investigated further.

## Conclusion

In this study, the EaMIC3 receptor molecule, UBE2F, was identified by YTH, and the molecular characterization of UBE2F was analyzed. All the results imply that EaMIC3 and the receptor protein UBE2F might be the target molecules involved in *E. acervulina* invasion.

## Abbreviations

*E. acervulina*: *Eimeria acervulina*
MIC3: Microneme 3
EaMIC3: MIC3 protein of *E. acervulina*
UBE2F: Ubiquitin conjugating enzyme E2F
rUBE2F: Recombinant protein of UBE2F
ORF: Opening reading frame
YTH: Yeast two-hybrid
BWGs: Average body weight gains
cAKP: Cell alkaline phosphatase
DAB: 3, 30-diaminobenzidine
OPG: Oocysts per gram feces
ANOVA: One-way analysis of variance

## References

[1] Augustine PC. 2001 Cell: sporozoite interactions and invasion by apicomplexan parasites of the genus *Eimeria*. International Journal for Parasitology, 31(1), 1–8. 11286188 10.1016/s0020-7519(00)00150-8

[2] Bakos G, Yu L, Gak IA, Roumeliotis TI, Liakopoulos D, Choudhary JS, Mansfeld J. 2018 An E2-ubiquitin thioester-driven approach to identify substrates modified with ubiquitin and ubiquitin-like molecules. Nature Communications, 9(1), 4776. 10.1038/s41467-018-07251-5 PMC6235928 30429481

[3] Barreau C, Touray M, Pimenta PF, Miller LH, Vernick KD. 1995 *Plasmodium gallinaceum*: sporozoite invasion of *Aedes aegypti* salivary glands is inhibited by anti-gland antibodies and by lectins. Experimental Parasitology, 81(3), 332–343. 7498430 10.1006/expr.1995.1124

[4] Brennan JDG, Kent M, Dhar R, Fujioka H, Kumar N. 2000 *Anopheles gambiae* salivary gland proteins as putative targets for blocking transmission of malaria parasites. Proceedings of the National Academy of Sciences of the United States of America, 97(25), 13859–13864. 11087838 10.1073/pnas.250472597 PMC17666

[5] Carruthers VB, Tomley FM. 2008 Microneme proteins in apicomplexans. Sub-Cellular Biochemistry, 47, 33–45. 18512339 10.1007/978-0-387-78267-6_2 PMC2847500

[6] Chien CT, Bartel PL, Sternglanz R, Fields S. 1991 The two-hybrid system: a method to identify and clone genes for proteins that interact with a protein of interest. Proceedings of the National Academy of Sciences of the United States of America, 88(21), 9578–9582. 1946372 10.1073/pnas.88.21.9578 PMC52761

[7] Cornelissen JB, Swinkels WJ, Boersma WA, Rebel JM. 2009 Host response to simultaneous infections with *Eimeria acervulina*, *maxima* and *tenella*: a cumulation of single responses. Veterinary Parasitology, 162(1–2), 58–66. 19272712 10.1016/j.vetpar.2009.02.001

[8] Crane MS, Murray PK, Gnozzio MJ, MacDonald TT. 1988 Passive protection of chickens against *Eimeria tenella* infection by monoclonal antibody. Infection & Immunity, 56(4), 972–976. 3346078 10.1128/iai.56.4.972-976.1988 PMC259400

[9] Elsasser TH, Miska K, Kahl S, Fetterer RH, Martinez Ramirez A. 2018 Temporal pattern changes in duodenal protein tyrosine nitration events in response to *Eimeria acervulina* infection in chickens. Journal of Animal Science, 96(6), 2125–2138. 29688400 10.1093/jas/sky140 PMC6095395

[10] Fatoba AJ, Adeleke MA. 2018 Diagnosis and control of chicken coccidiosis: a recent update. Journal of Parasitic Diseases, 42(4), 483–493. 30538344 10.1007/s12639-018-1048-1 PMC6261147

[11] Feng T, Deng L, Lu X, Pan W, Wu Q, Dai J. 2018 Ubiquitin-conjugating enzyme UBE2J1 negatively modulates interferon pathway and promotes RNA virus infection. Virology Journal, 15(1), 132. 30157886 10.1186/s12985-018-1040-5 PMC6114777

[12] Galli GM, Baldissera MD, Griss LG, Souza CF, Fortuoso BF, Boiago MM, Gris A, Mendes RE, Stefani LM, da Silva AS. 2019 Intestinal injury caused by *Eimeria* spp. impairs the phosphotransfer network and gain weight in experimentally infected chicken chicks. Parasitology Research, 118(5), 1573–1579. 30815727 10.1007/s00436-019-06221-0

[13] Huang J, Liu T, Li K, Song X, Yan R, Xu L, Li X. 2018 Proteomic analysis of protein interactions between *Eimeria maxima* sporozoites and chicken jejunal epithelial cells by shotgun LC-MS/MS. Parasites & Vectors, 11(1), 226. 29618377 10.1186/s13071-018-2818-4 PMC5885459

[14] Huang X, Liu J, Tian D, Li W, Zhou Z, Huang J, Song X, Xu L, Yan R, Li X. 2018 The molecular characterization and protective efficacy of microneme 3 of *Eimeria mitis* in chickens. Veterinary Parasitology, 258, 114–123. 30105971 10.1016/j.vetpar.2018.06.020

[15] Jeong DE, Lee Y, Lee SJV. 2018 Western blot analysis of *C. elegans* proteins, in Hypoxia. Methods in Molecular Biology, Vol. 1742, Huang L, Editor. Humana Press: New York, NY. 10.1007/978-1-4939-7665-2_19 29330803

[16] Jin B, Wang J, Liu X, Fang S, Jiang B, Hofmann K, Yin J, Zhao B. 2018 Ubiquitin-mimicking peptides transfer differentiates by E1 and E2 enzymes. Biomed Research International, 2018, 6062520. 30246024 10.1155/2018/6062520 PMC6136576

[17] Joyner LP, Long PL. 1974 The specific characters of the *Eimeria*, with special reference to the coccidia of the fowl. Avian Pathology, 3(3), 145–157. 18777269 10.1080/03079457409353827

[18] Klotz C, Gehre F, Lucius R, Pogonka T. 2007 Identification of *Eimeria tenella* genes encoding for secretory proteins and evaluation of candidates by DNA immunisation studies in chickens. Vaccine, 25(36), 6625–6634. 17675183 10.1016/j.vaccine.2007.06.048

[19] Labbe M, de Venevelles P, Girard-Misguich F, Bourdieu C, Guillaume A, Pery P. 2005 *Eimeria tenella* microneme protein EtMIC3: identification, localisation and role in host cell infection. Molecular and Biochemical Parasitology, 140(1), 43–53. 15694485 10.1016/j.molbiopara.2004.12.002

[20] Lai L, Simpson P, Bumstead J, Tomley F, Matthews S. 2009 Complete NMR assignments for the second microneme adhesive repeat (MAR) domain from *Eimeria tenella* microneme protein EtMIC3. Biomolecular NMR Assignments, 3(2), 175–177. 19888684 10.1007/s12104-009-9168-2

[21] Lai L, Bumstead J, Liu Y, Garnett J, Campanero-Rhodes MA, Blake DP, Palma AS, Chai W, Ferguson DJ, Simpson P, Feizi T, Tomley FM, Matthews S. 2011 The role of sialyl glycan recognition in host tissue tropism of the avian parasite *Eimeria tenella*. PLoS Pathogens, 7(10), e1002296. 22022267 10.1371/journal.ppat.1002296 PMC3192848

[22] Liu J, Liu L, Li L, Tian D, Li W, Xu L, Yan R, Li X, Song X. 2018 Protective immunity induced by *Eimeria* common antigen 14-3-3 against *Eimeria tenella*, *Eimeria acervulina* and *Eimeria maxima*. BMC Veterinary Research, 14(1), 337. 30419898 10.1186/s12917-018-1665-z PMC6233286

[23] Long PL, Millard BJ. 1976 Studies on site finding and site specificity of *Eimeria praecox*, *Eimeria maxima* and *Eimeria acervulina* in chickens. Parasitology, 73(3), 327–336. 1012749 10.1017/s0031182000047004

[24] Lv Z, Williams KM, Yuan L, Atkison JH, Olsen SK. 2018 Crystal structure of a human ubiquitin E1-ubiquitin complex reveals conserved functional elements essential for activity. Journal of Biological Chemistry, 293(47), 18337–18352. 30279270 10.1074/jbc.RA118.003975 PMC6254350

[25] Marugan-Hernandez V, Fiddy R, Nurse-Francis J, Smith O, Pritchard L, Tomley FM. 2017 Characterization of novel microneme adhesive repeats (MAR) in *Eimeria tenella*. Parasites & Vectors, 10(1), 491. 29041988 10.1186/s13071-017-2454-4 PMC5646145

[26] Metzger MB, Pruneda JN, Klevit RE, Weissman AM. 2014 RING-type E3 ligases: master manipulators of E2 ubiquitin-conjugating enzymes and ubiquitination. Biochimica et Biophysica Acta, 1843(1), 47–60. 23747565 10.1016/j.bbamcr.2013.05.026 PMC4109693

[27] Pasupala N, Morrow ME, Que LT, Malynn BA, Ma A, Wolberger C. 2018 OTUB1 non-catalytically stabilizes the E2 ubiquitin-conjugating enzyme UBE2E1 by preventing its autoubiquitination. Journal of Biological Chemistry, 293(47), 18285–18295. 30282802 10.1074/jbc.RA118.004677 PMC6254341

[28] Rose ME, Hesketh P. 1991 *Eimeria tenella*: localization of the sporozoites in the caecum of the domestic fowl. Parasitology, 102(Pt 3), 317–324. 1830957 10.1017/s0031182000064258

[29] Spiro RG. 2002 Protein glycosylation: nature, distribution, enzymatic formation, and disease implications of glycopeptide bonds. Glycobiology, 12(4), 43R–56R. 10.1093/glycob/12.4.43r 12042244

[30] Upadhaya SD, Cho SH, Chung TK, Kim IH. 2019 Anti-coccidial effect of essential oil blends and vitamin D on broiler chickens vaccinated with purified mixture of coccidian oocyst from *Eimeria tenella* and *Eimeria maxima*. Poultry Science, 98(7), 2919–2926. 10.3382/ps/pez040 30778571

[31] Wallach M, Pillemer G, Yarus S, Halabi A, Mencher D. 1990 Passive immunization of chickens against *Eimeria maxima* infection with a monoclonal antibody developed against a gametocyte antigen. Infection & Immunity, 58(2), 557–562. 2298492 10.1128/iai.58.2.557-562.1990 PMC258493

[32] Yang WC, Yang CY, Liang YC, Yang CW, Li WQ, Chung CY, Yang MT, Kuo TF, Lin CF, Liang CL, Chang CL. 2019 Anti-coccidial properties and mechanisms of an edible herb, *Bidens pilosa*, and its active compounds for coccidiosis. Scientific Reports, 9(1), 2896. 30814608 10.1038/s41598-019-39194-2 PMC6393484

[33] Zhang Z, Huang J, Li M, Sui Y, Wang S, Liu L, Xu L, Yan R, Song X, Li X. 2014 Identification and molecular characterization of microneme 5 of *Eimeria acervulina*. PloS One, 9(12), e115411. 25531898 10.1371/journal.pone.0115411 PMC4274027

[34] Zhang Z, Wang S, Huang J, Liu L, Lu M, Li M, Sui Y, Xu L, Yan R, Song X, Li X. 2015 Proteomic analysis of *Eimeria acervulina* sporozoite proteins interaction with duodenal epithelial cells by shotgun LC-MS/MS. Molecular and Biochemical Parasitology, 202(2), 29–33. 10.1016/j.molbiopara.2015.09.006 26439303

[35] Zhang Z, Liu L, Huang J, Wang S, Lu M, Song X, Xu L, Yan R, Li X. 2016 The molecular characterization and immune protection of microneme 2 of *Eimeria acervulina*. Veterinary Parasitology, 215, 96–105. 26790744 10.1016/j.vetpar.2015.10.028

[36] Zhang Z, Liu X, Yang X, Liu L, Wang S, Lu M, Ehsan M, Gadahi JA, Song X, Xu L, Yan R, Li X. 2016 The molecular characterization and immunity identification of microneme 3 of *Eimeria acervulina*. Journal of Eukaryotic Microbiology, 63(6), 709–721. 27037629 10.1111/jeu.12318

[37] Zhang Z, Wang S, Li C, Liu L. 2017 Immunoproteomic analysis of the protein repertoire of unsporulated *Eimeria tenella* oocysts. Parasite, 24, 48. 29194033 10.1051/parasite/2017047 PMC5711376

